# Supplementary material for: Analysis of Bone Histomorphometry in Rat and Guinea Pig Animal Models Subject to Hypoxia
Source: Int J Mol Sci. 2022 Oct 22;23(21):12742. doi: 10.3390/ijms232112742 (PMC9655516; doi:10.3390/ijms232112742)
Supplement: Supplementary file 1 [file ijms-23-12742-s001.zip › ijms-1971332-supplementary.pdf]

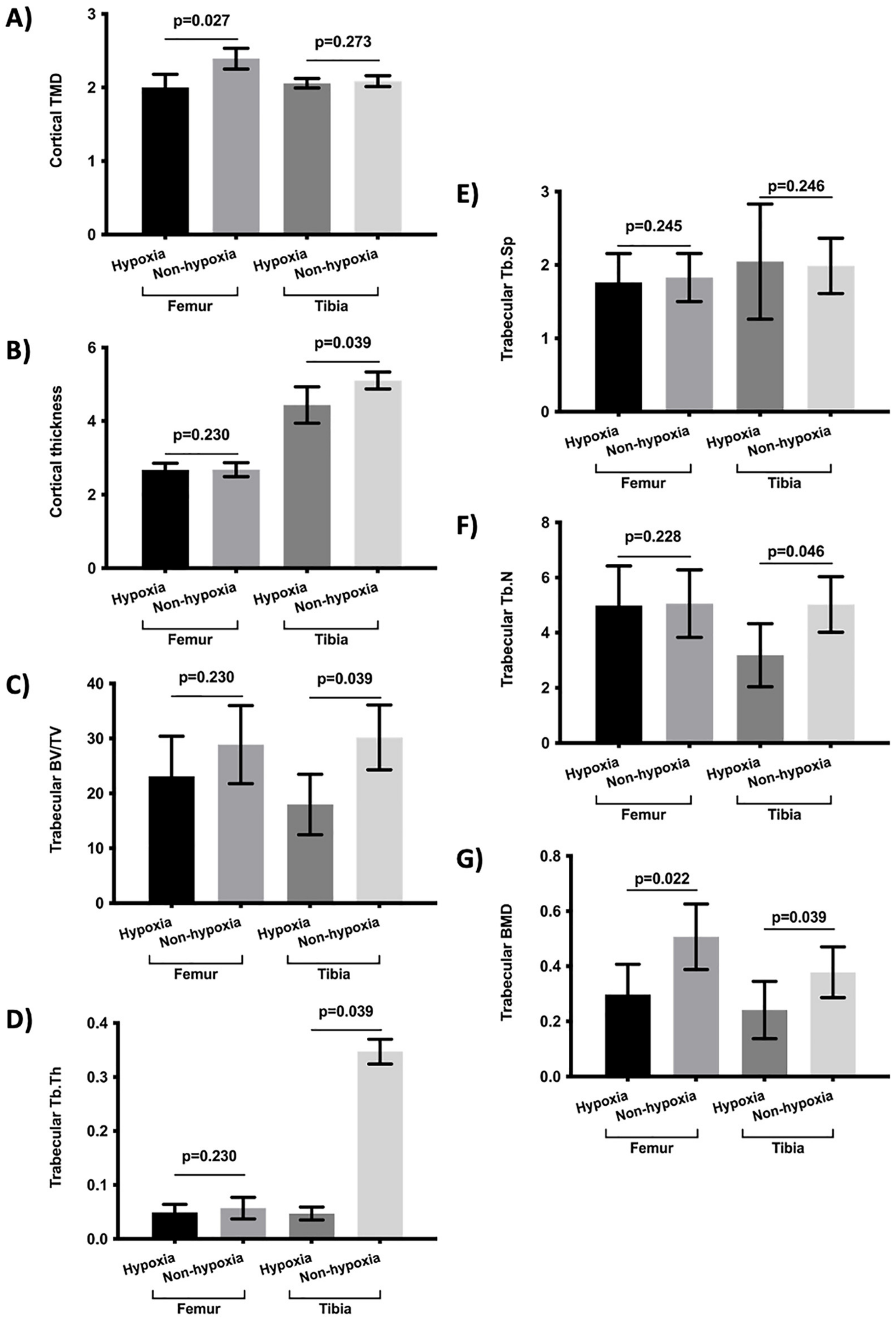

Supplementary figure S1. Comparison of bone morphometry parameters between rats subject to hypoxic conditions and controls (non-hypoxia). (A) Cortical TMD, (B) Cortical thickness, (C) Trabecular BV/TV, (D) Tb.Th, (E) Tb.Sp, (F) Tb. N and (G) Trabecular BMD. Variables are presented as mean (standard deviation). TMD: tissue mineral density, BV/TV: percent bone volume; Tb.Th: Trabecular thickness; Tb.Sp: Trabecular separation; Tb.N: Trabecular number; BMD: bone mineral density.

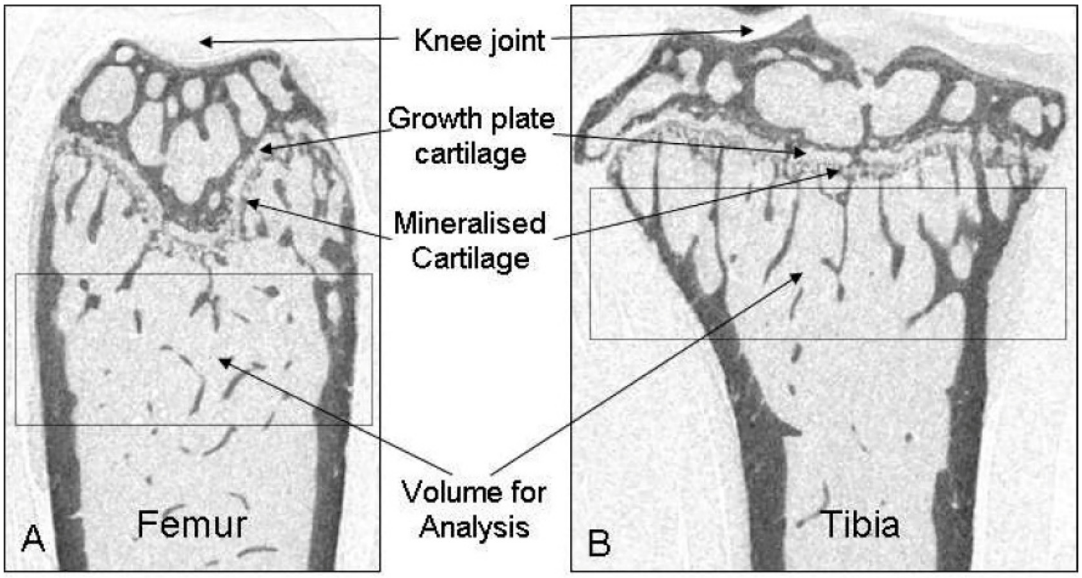

Supplementary figure S2. Definition of histomorphometry area analyzed. Cortical and trabecular areas of the tibia and femur were studied. The trabecular bone analysis was performed in the distal femur and proximal tibia areas. For this, the regions of interest included the 2 mm region from the growth plate. For analysis of the cortical area, the central regions of the femur and tibia were selected, comprising 10-15 % of the sample length.

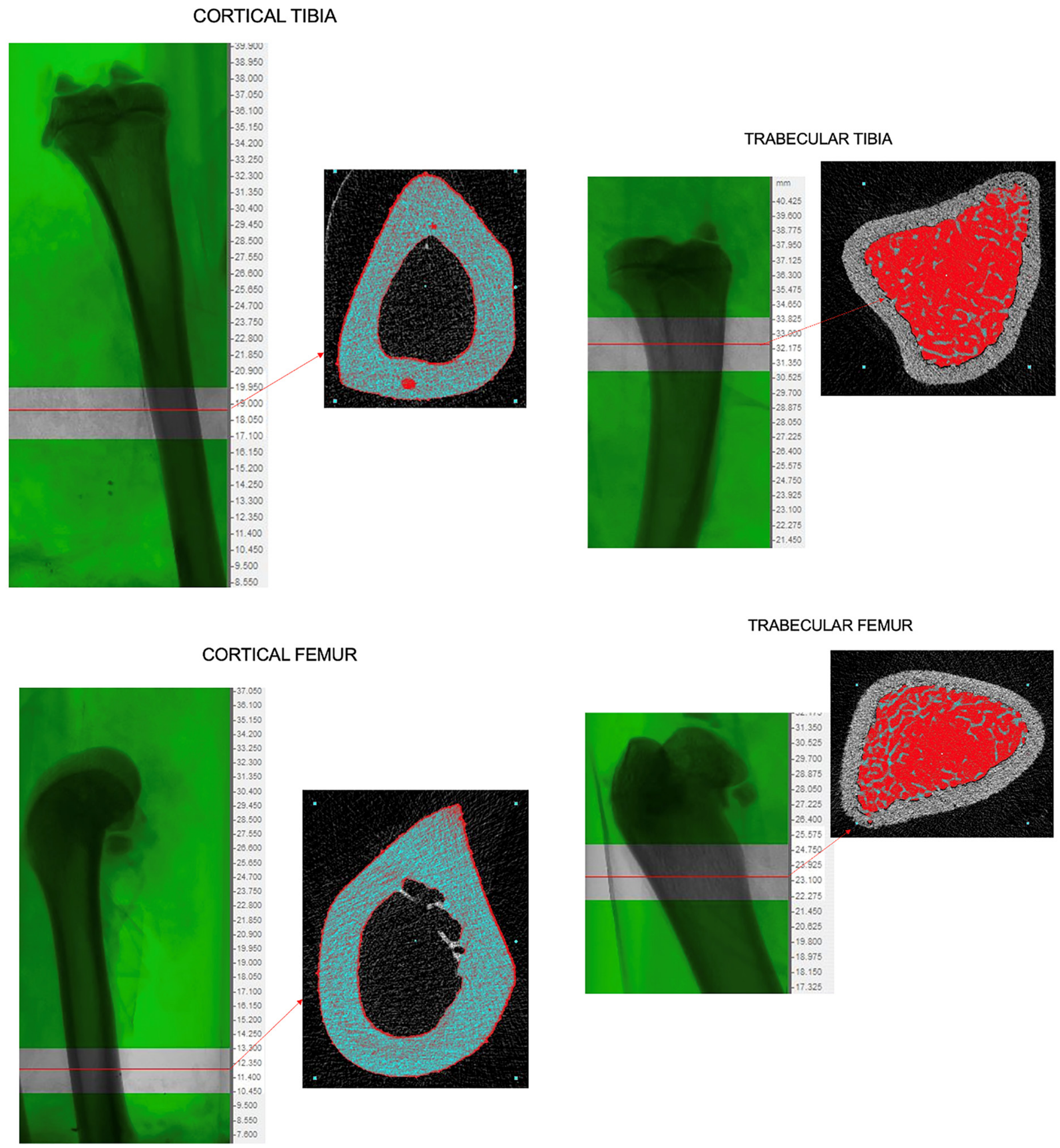

Supplementary figure S3. Examples of trabecular and cortical analysed regions.

Supplementary table S1. Scan parameters

Variable	Standard unit	Value
Voxel size	µm3	300.76
Source Voltage	kV	50
Source Current	µA	100
Exposure time	ms	4920
Frame averaging	N	5
Projections	N	130
